# Integrative Transcriptomics and Metabolomics Reveal the Key Metabolic Pathways in Endophyte-Infected Rice Seedlings Resistance to Na_2_CO_3_ Stress

**DOI:** 10.3390/plants14101524

**Published:** 2025-05-19

**Authors:** Xinnan Wang, Yanan Li, Hefei Sun, Lihong Zhang, Xuemei Li

**Affiliations:** 1 College of Life Science, Shenyang Normal University, Shenyang 110034, China; wangxinnan0917@163.com (X.W.); 15042129995@163.com (Y.L.); sunhefei99@163.com (H.S.); 2 School of Environmental Science, Liaoning University, Shenyang 110036, China

**Keywords:** *Oryza sativa* L., Na_2_CO_3_ stress, endophyte-plant interaction, RNA-seq, GC-MS analysis

## Abstract

Soil saline-alkalization is a key factor affecting rice growth and physiological metabolism, which leads to reduced yields. Endophyte EF0801 significantly promoted growth and improved its saline-alkali resistance. We investigated growth parameters and physiological indices of endophyte EF0801-infected and control rice seedlings under sodium carbonate (Na_2_CO_3_) stress. The results showed that endophyte-infected rice seedlings showed plant height increase by 1.25-fold, root length shortening by 0.79-fold, sucrose synthase (SS), sucrose phosphosynthase (SPS), hexokinase (HXK), and α-glucosidase (α-GC) activities increased by 0.15-fold, 0.29-fold, 0.06-fold, and 1.45-fold, respectively, and β-glucosidase (β-GC) activity decreased by 0.12-fold. Utilizing gas chromatography and mass spectrometry (GC-MS) technology and RNA sequencing (RNA-seq) technology, we identified 419 differentially expressed genes (DEGs) and 37 differentially accumulated metabolites (DAMs). Comprehensive enrichment analysis of DAMs and DEGs showed that 6 DEGs and 6 DAMs were strongly correlated with the mitigating effects of endophytes on rice leaves under Na_2_CO_3_ treatment, highlighting the co-enrichment in starch and sucrose metabolism, as well as alanine, aspartate, and glutamate metabolism. The gene encoding HXK was found to be upregulated in endophyte-infected rice seedlings under Na_2_CO_3_ stress. HXK plays a key role in the conversion of fructose and glucose to fructose 6-phosphate (F-6-P) and glucose 6-phosphate (G-6-P), which are important intermediates in cellular energy metabolism and glycolytic pathways, providing energy and biosynthesis of precursor substances. Our findings provide a potential perspective for unraveling the molecular response of endophyte-mediated saline-alkali resistance in rice leaves and a theoretical rationale for exploring the mechanisms of growth-promoting effects by endophytes.

## 1. Introduction

Soil saline-alkalization critically affects global food crop growth and production [1]. Reports indicate that an estimated 113 million hectares of land are affected by salinization globally, about 25% of the global land area [2,3]. Generally, saline-alkali causes osmotic stress and high pH in plants and disrupts ionic balance [4,5], ultimately affecting plant growth and production [6,7]. Rice, the cereal crop with the widest cultivation area in China, is particularly sensitive to saline-alkali, especially at its seedling stage and growth period [8,9]. Therefore, it is necessary to study the damage caused by Na_2_CO_3_ treatment to plants to improve their resistance to saline-alkali.

Endophytes are microorganisms that live in normally developing plant organs and tissues and do not negatively affect the host [10]. Numerous studies have found that endophytes can promote plant nutrient uptake [11,12], enhance defense-related gene expression [13], increase endogenous hormone levels [14], and positively alter related metabolite production [15] under abiotic stress. Based on previous studies, it was found that a fungal endophyte EF0801 significantly increased the stability of photosystem II in rice seedlings [16], regulated mineral element accumulation and organic acid metabolism [17], and increased antioxidant enzyme activities [18] under Na_2_CO_3_ treatment.

Metabolomes are used to observe a variety of metabolites in plants at specific times and environments [19], which is important for studying plant metabolism pathways and improving plant response to adverse stress [20]. Currently, metabolomics has been applied to study the reaction of barley to Na_2_CO_3_ treatment under endophyte infection [21]. The transcriptome is used to analyze plant gene expression levels [22] and alleviation mechanisms of endophytes in rice seedlings under Na_2_CO_3_ treatment [23]. However, integrative transcriptomic and metabolomic analyses provide a novel and formidable tool for elucidating the relationship between genotype and phenotype. Li et al. [24] demonstrated how ecological adaptations develop in two distinct ecotypes of wild soybeans by examining gene expression and metabolic variations in a thorough and detailed study of the transcriptome and metabolome. Shao et al. [25] combined transcriptomic and metabolomic analyses to analyze the effects of the biocontrol endophyte R31 on sweet corn and found that DEGs and DAMs were co-enriched for flavonoid biosynthesis. Therefore, we performed metabolomics and transcriptomics of endophyte-infected rice leaves under saline-alkali stress using GC-MS and RNA-Seq to identify and screen differentially accumulated metabolites (DAMs) and differentially expressed genes (DEGs). The common key metabolic pathways of DAMs and DEGs were further explored through co-enrichment analysis. This study focuses on revealing the mechanism of enhancing saline-alkali stress tolerance in rice seedlings by endophytes at the molecular level, screening potential resistance genes and metabolites, and providing theoretical support for agricultural production and scientific research.

## 2. Results

### 2.1. Growth Indexes of Rice Seedlings

**Figure 1:**
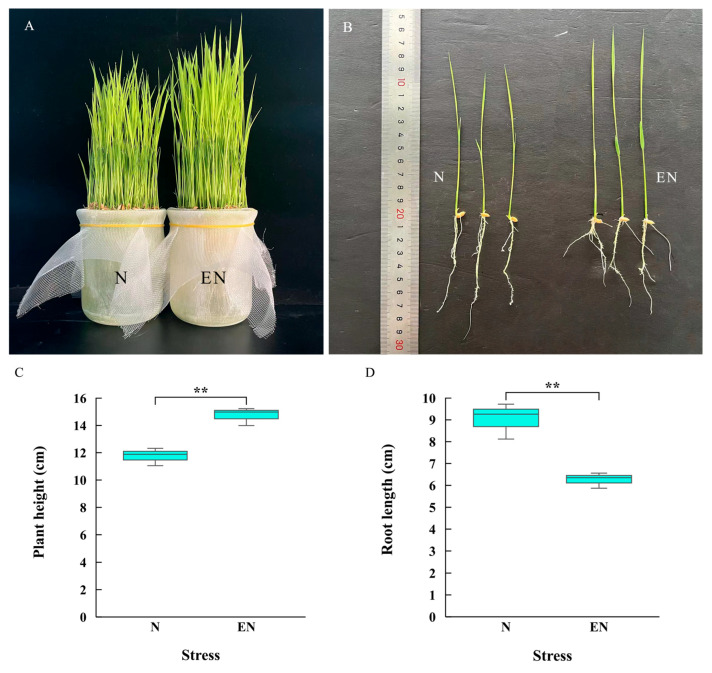
The growth of endophyte-infected and control rice seedlings under Na_2_CO_3_ stress. Phenotypes (**A**,**B**), plant height (**C**), and root length (**D**). ** indicated the significant differences of *p* < 0.01.

After 7 d of treatment, significant differences in phenotype and growth were observed between endophyte-infected and control rice seedlings (Figure 1A,B). Compared with control rice seedlings, endophyte-infected rice seedlings showed that plant height increased by 1.25-fold (Figure 1C) and root length decreased by 0.79-fold (Figure 1D).

### 2.2. Changes and Validation of Sucrose Metabolism-Related Enzyme Activities

**Figure 2:**
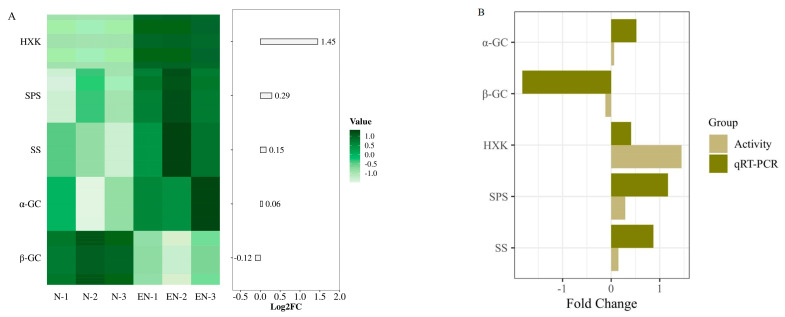
Changes in activities and expressions of sucrose metabolism-related enzymes in endophyte-infected versus control rice seedlings under Na_2_CO_3_ stress. Heatmap analysis of sucrose metabolism-related enzyme activities in endophyte-infected versus control rice seedlings under Na_2_CO_3_ stress (**A**). Bar graphs were plotted using log_2_FC values of enzyme activity and qRT-PCR relative expression for fold change comparison (**B**).

Under Na_2_CO_3_ treatment, sucrose synthase (SS), sucrose phosphate synthase (SPS), α-Glucosidase (α-GC), and hexokinase (HXK) activities were increased by 0.15-fold, 0.29-fold, 0.06-fold, and 1.45-fold, respectively, and β-Glucosidase (β-GC) was decreased by 0.12-fold in rice leaves inoculated with endophytes (Figure 2A). To test the accuracy of sucrose metabolism-related enzymes’ (SS, SPS, HXK, α-GC, and β-GC) activity data, qRT-PCR was performed to verify the enzyme-regulated related genes. The validation results showed that the trend of sucrose metabolism-related enzyme activities was consistent with the qRT-PCR validation results (Figure 2B).

### 2.3. Analysis and Validation of RNA-Seq Data

**Figure 3:**
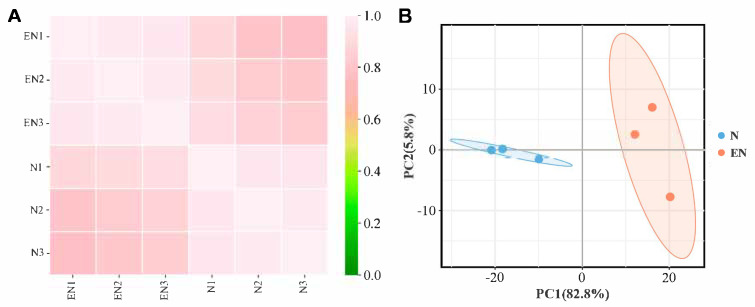
RNA-Seq analysis of endophyte-infected versus control rice seedlings under Na_2_CO_3_ stress. Spearman correlation analysis (**A**) and PCA (**B**).

**Figure 4:**
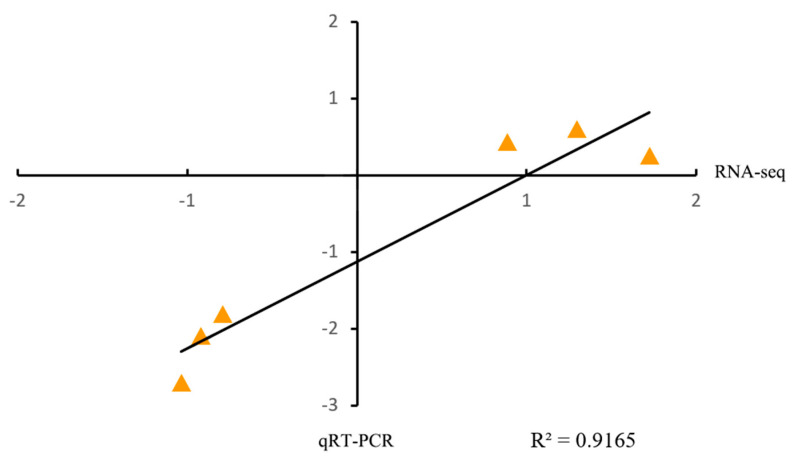
Validation of the expression level of genes from RNA-seq using qRT-PCR. A comparison of fold change (FC) was done using scatter plots using log_2_FC values obtained from RNA-seq and qRT-PCR.

Clean reads of 44.32 gigabytes (Gb) were obtained for six cDNA libraries. The sample Q30% was more than 93.87%, and the GC content was between 50.76% and 51.88%. To check transcriptome data similarity and credibility, we performed Spearman correlation analysis and PCA on the sequencing data, and the results obtained suggested that the data were reliable and ready for subsequent analysis (Figure 3A,B). To further determine the RNA-seq data reliability, we randomly picked six genes to perform qRT-PCR validation. The validation results showed a consistent trend between RNA-seq data and qRT-PCR validation (R^2^ = 0.9165) (Figure 4).

### 2.4. Screening and Functional Analysis of DEGs

**Figure 5:**
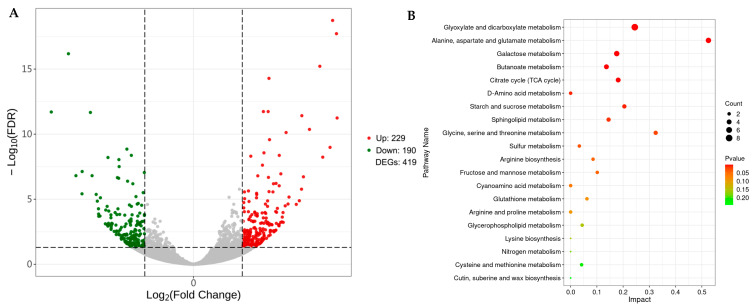
Transcriptome analysis of endophyte-infected versus control rice seedlings under Na_2_CO_3_ stress. Volcano plot of DEGs (**A**) and KEGG enrichment analysis (**B**). Significantly up- and down-regulated genes are indicated by red and green dots, respectively.

Based on the FDR < 0.05 and |log_2_FC| ≥ 0.585 of the screened DEGs, 419 DEGs were detected, including 229 up- and 190 down-regulated DEGs (Figure 5A). KEGG enrichment showed that 69 DEGs were enriched in 53 metabolic pathways (Table S2). Among them, eight significantly enriched pathways were starch and sucrose metabolism (ko00500), monoterpenoid biosynthesis (ko00902), cysteine and methionine metabolism (ko00270), nitrogen metabolism (ko00910), galactose metabolism (ko00052), cyanoamino acid metabolism (ko00460), carotenoid biosynthesis (ko00906) and alanine, aspartate, and glutamate metabolism (ko00250) (Figure 5B).

### 2.5. Assessment and Bioinformatics Analysis of DAMs

**Figure 6:**
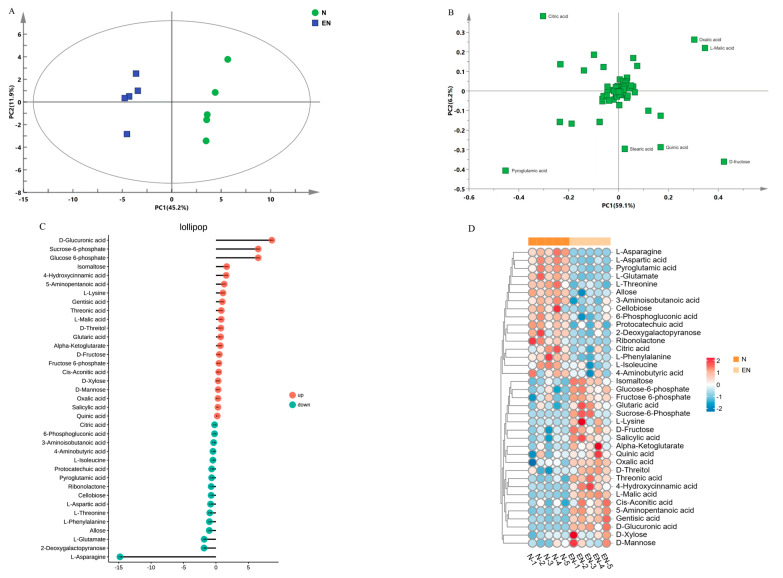
Metabolome analysis of endophyte-infected versus control rice seedlings under Na_2_CO_3_ stress. PCA (**A**), PLS-DA (**B**), fold changes (**C**), and hierarchical clustering heat map (**D**).

In this experiment, we analyzed the metabolites of endophyte-infected and control rice seedlings under Na_2_CO_3_ treatment. PC1 (first principal component) was 45.2%, and PC2 (second principal component) was 11.9%, and the results showed significantly different data for the two treatment groups (Figure 6A). PLS-DA results showed that components contributing to PC1 were oxalic acid, l-malic acid, pyroglutamic acid, and d-fructose; components contributing to PC2 were citric acid, quinic acid, and stearic acid (Figure 6B, Table S3). Screening of metabolites according to VIP > 1 and *p* < 0.05 revealed 37 metabolites with significant changes between the N and EN groups, including 10 amino acids, 6 glycolysis and TCA cycles, 9 organic acids, 12 sugars and polyols (Table 1). Fold change analysis showed that 21 and 16 DAMs were up- and down-regulated (Figure 6C). Hierarchical clustering analysis could more directly show the fold change of metabolites between E and EN groups (Figure 6D).

**Figure 7:**
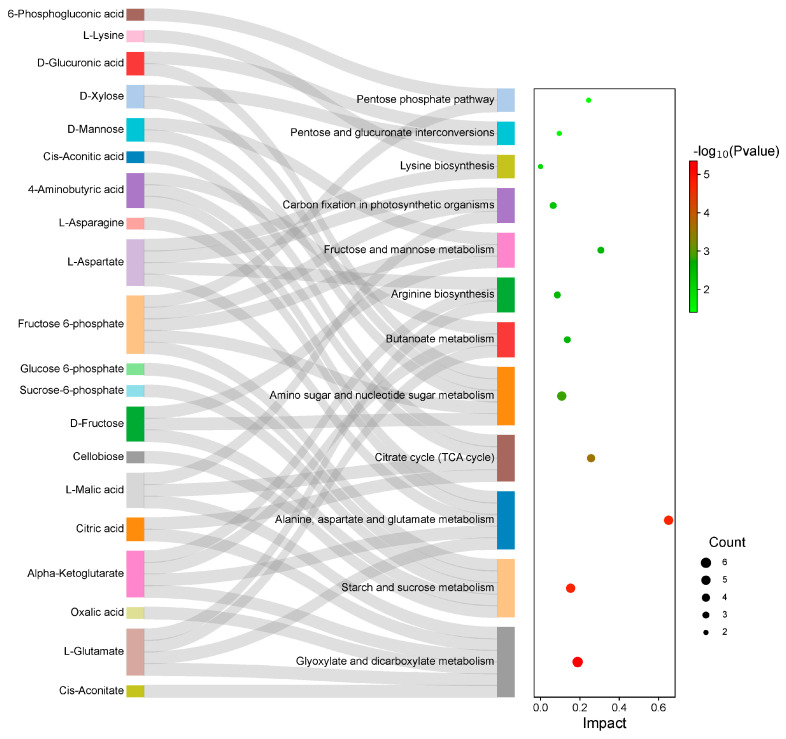
KEGG enrichment analysis of endophyte-infected versus control rice seedlings under Na_2_CO_3_ stress.

To elucidate the role of DAMs in the N and EN groups, KEGG pathway analysis was performed on DAMs. The results show that 38 metabolic pathways were enriched and 12 metabolic pathways were significantly enriched (Table S4). Among them, DAMs were most enriched in carbohydrate- and amino acid-related metabolic pathways (Figure 7).

### 2.6. Integrated Transcriptome and Metabolome Analysis

**Figure 8:**
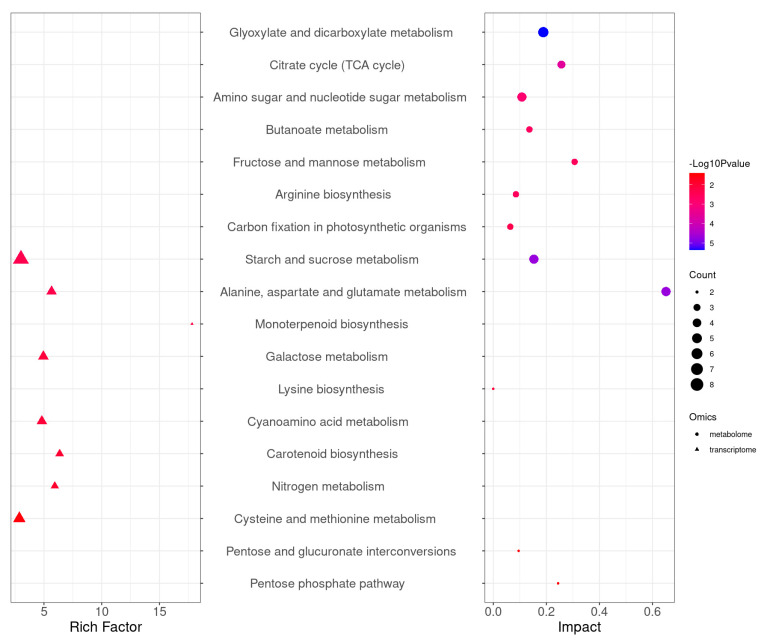
Integrated analysis of transcriptome and metabolome pathway enrichment in endophyte-infected versus control rice seedlings under Na_2_CO_3_ stress.

**Figure 9:**
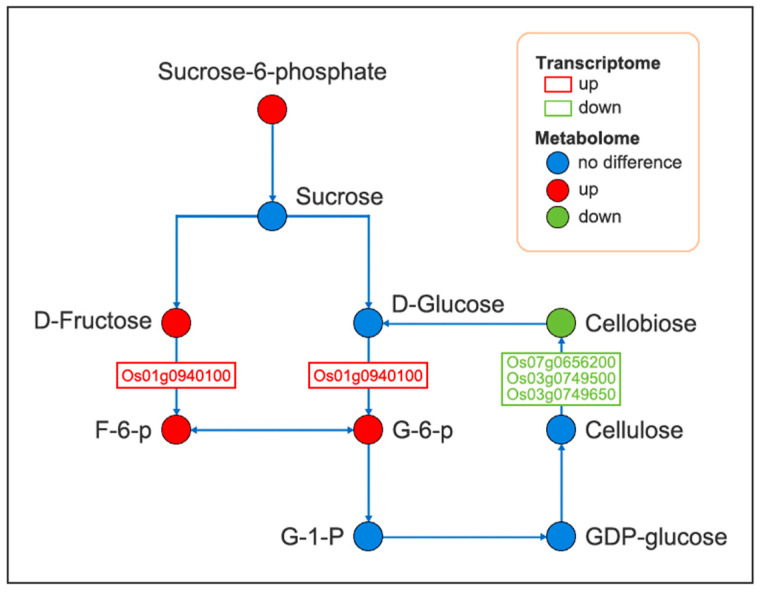
Changes in DAMs and DEGs in starch and sucrose metabolism pathways in endophyte-infected versus control rice seedlings under Na_2_CO_3_ stress.

**Figure 10:**
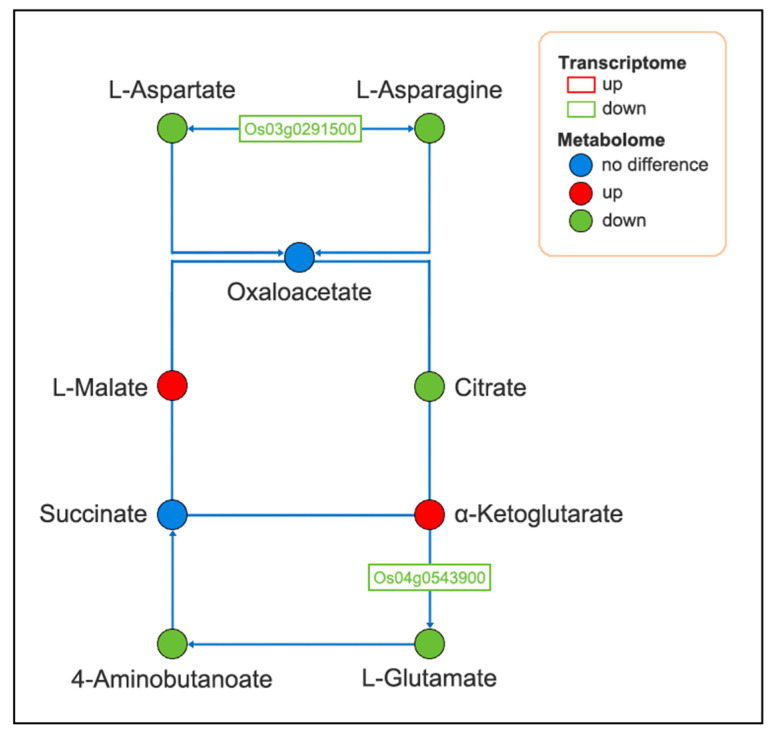
Changes in DAMs and DEGs in alanine, aspartate, and glutamate metabolism pathways in endophyte-infected versus control rice seedlings under Na_2_CO_3_ stress.

To study the metabolic changes in endophyte-infected and control rice under Na_2_CO_3_ treatment, we performed KEGG joint enrichment analysis of DEGs and DAMs. Data indicated that the co-enrichment pathways were starch and sucrose metabolism as well as alanine, aspartate, and glutamate metabolism pathways (Figure 8). Construction of the gene and metabolite networks of starch and sucrose metabolic pathways revealed that the gene encoding hexokinase (HXK) [EC:2.7.1.1] (*Os01g0940100*) was highly correlated with f-6-p and g-6-p; the genes encoding α-glucosidase (α-GC) [EC:3.2.1.20] (*Os07g0656200*, *Os03g0749500*, and *Os03g0749650*) were positively correlated with cellobiose (Figure 9). Construction of gene and metabolite networks for alanine, aspartate, and glutamate metabolic pathways revealed that the gene encoding asparagine synthase (ASNS) [EC: 6.3.5.4] (*Os03g0291500*) was positively correlated with l-aspartic acid and l-asparagine; the gene encoding glutamate dehydrogenase (GDH) [EC. 1.4.1.3] gene (*Os04g0543900*) was positively correlated with l-glutamate and pyroglutamic acid (Figure 10). The final six genes were found to be associated with starch and sucrose metabolic pathways and alanine, aspartate, and glutamate metabolic pathways. It was demonstrated that starch and sucrose metabolic pathways and alanine, aspartate, and glutamate metabolic pathways are crucial in the mechanism of endophyte-mediated saline-alkali resistance in rice.

## 3. Discussion

Soil saline-alkalization markedly affects rice growth, development, and ultimately yields. Therefore, improving rice tolerance to sustain rice production requires a comprehensive characterization of the multiple biological processes under Na_2_CO_3_ treatment. Analysis of endophyte-host interactions revealed that the endophyte significantly promotes plant growth and maintains homeostatic balance [26,27], and based on our previous studies [28], we found that endophyte infection significantly increased the mineral element content of rice seedlings. Studies found that rice seedlings were significantly taller but had reduced root length after endophyte infection compared to the Na_2_CO_3_-treated group. Consistent with our previous descriptions [16,18], rice seedlings were sensitive to Na_2_CO_3_ treatment, and endophyte infection resulted in significant changes in phenotype and growth parameters. Wu et al. [27] found that inoculation with the dark septate endophyte S16 promoted sweet cherry growth and development.

Genes regulate various metabolic processes in plants, gene expression changes affect metabolite levels to some extent, and these metabolites are associated with potential enzymes or genes that can be directly involved in plant physiological, biochemical, and metabolic processes [29]. In this study, KEGG co-enrichment analysis of screened DAMs and DEGs by integrating transcriptomic and metabolomic data revealed starch and sucrose metabolism, as well as alanine, aspartate, and glutamate metabolism as co-enrichment pathways.

Starch and sucrose metabolism are the main carbohydrate metabolism pathways in plants, providing an adequate carbon source for plant growth and development, whereas carbohydrates, as the main products of photosynthesis, not only provide energy for plant growth but also regulate osmotic homeostasis thus maintaining the dynamic balance of cells under various stress states [30,31,32,33]. In addition, carbohydrates are key molecules and reactants that regulate various metabolic processes [34]. It was found that five DAMs (including f-6-p, g-6-p, sucrose-6-phosphate, fructose, and cellobiose) were involved in the starch and sucrose metabolism pathway. In this study, we determined the activities of SS, SPS, HXK, α-GC, and β-GC in endophyte-infected and control rice seedlings under Na_2_CO_3_ treatment. SS and SPS are the main enzymes involved in sucrose metabolism, and they catalyze the production of UDP-glucose, fructose, and sucrose-6-phosphate, respectively [35]. α-GC, also known as α-d-glucoside hydrolase, is a carbohydrate-digesting enzyme that catalyzes the hydrolysis of α-1,4-glycosidic bonds, or transfers free glucose residues to another carbohydrate substrate to form α-1,6-glycosidic bonds and form isomaltose, while β-GC is an enzyme that hydrolyzes β-glycosidic bonds and participates in the hydrolysis of cellobiose [17,36]. HXK catalyzes the production of f-6-p from fructose, which is further phosphorylated during glycolysis by phosphofructokinase, and glucose is catalyzed by HXK to produce g-6-p, which is essential for polysaccharide biosynthesis [37], as well as providing sufficient substrates for a variety of metabolic pathways.

Metabolome data showed that the majority of DAMs participating in starch and sucrose metabolism were up-regulated, implying that endophyte-infected rice seedlings mitigated the inhibitory effect of saline-alkali by accumulating carbohydrates. Richardson et al. [38] found that the content of carbohydrates in fungal endophyte- (*Acremonium coenophialum* Morgan-Jones & Gains) infected leaves of tall fescue increased when grown under drought stress compared to uninfected tall fescue, which is consistent with the results of the present study. Combined with the metabolome data and sucrose metabolism-related enzyme activity assay data, the results showed that the increase of SS, SPS, HXK, and α-GC activities promoted the accumulation of fructose, g-6-p, f-6-p, and isomaltose, and the decrease of β-GC activity inhibited the synthesis of cellobiose, indicating that sucrose metabolism-related enzymes also actively participated in the synthesis and decomposition of metabolites in starch and sucrose metabolism pathways. Transcriptome data showed that β-GC and HXK were partially induced in endophyte-infected rice seedlings under Na_2_CO_3_ treatment, indicating activation of starch and sucrose metabolism. SkZ et al. [39] have found that maize seedlings infected with *Pseudomonas putida* showed a strengthened tolerance to drought treatment due to the ability of *Pseudomonas putida* to participate in the regulation of carbohydrate metabolic pathway genes. In the combined analysis of DEGs and DAMs, only f-6-p, g-6-p, and cellobiose content were detected. It was found that the expression of the gene encoding HXK was up-regulated with the accumulation of f-6-p and g-6-p, while the expression of the gene encoding β-GC was down-regulated with the decrease of cellobiose. In addition, combined transcriptome and metabolome data determined that the expression of DEGs involved in starch and sucrose metabolism followed the same trend as the content of DAMs.

Amino acid metabolism is one of the key pathways in plant response to a variety of abiotic stresses [40]. Amino acids are critical in modulating plant growth, physiological metabolism, maintaining intracellular osmoregulation, and protein structural integrity [41,42], and they are an important indicator for assessing crop tolerance to growth, development, and various stresses [43]. Alanine, aspartate, and glutamate metabolism plays important roles in participating in the regulation of phytohormones, thus directly or indirectly regulating crop growth and stress tolerance [44]. GDH acts as a reactive enzyme that catalyzes pyroglutamic acid generation from l-glutamate and assimilates NH4^+^-N [1]. ASNS is a metabolic enzyme that catalyzes the catabolism of glutamine and aspartate into asparagine [45]. Integration of transcriptome and metabolome data found that down-regulation expression of GDH (*Os04g0543900*) and ASNA *(Os03g0291500)* led to a decrease in the corresponding downstream products l-aspartate, l-glutamate, and l-asparagine, and a decrease in amino acid content, which may indicate that the presence of the endophyte altered the pathway of sugar synthesis and depletion, thereby affected the TCA cycle, changed the composition of the small-molecule osmotic-regulating substances in the plant from amino acids to sugar and sugar alcohols, and ultimately affected the pathway of energy production under Na_2_CO_3_ treatment. The results showed that the expression of relevant DEGs involved in the alanine, aspartate, and glutamate metabolism was consistent with the trend of DAMs content. Wang et al. [46] reported that genes involved in the regulation of amino acid metabolism were detected to be down-regulated in *Achnatherum inebrians* inoculated with *Epichloë gansuensis* under salt treatment, which is the same as the results of the present experiment.

Given that our results indicate that *Os04g0543900*, *Os03g0749500*, and *Os03g0749650*, which were screened under alanine, aspartate, and glutamate metabolic pathways and starch and sucrose metabolic pathways, have not yet been cloned from plants but may also be involved in the regulation of endophyte to alleviate Na_2_CO_3_ treatment in rice seedlings, there is a need for further research on these DEGs that are involved in plant–endophyte interactions.

Combining all the results of the studies, it was found that Na_2_CO_3_-treated rice seedlings showed some changes in gene expression and metabolite levels after endophyte infection; thus, the functions of these metabolites and genes are crucial for studying the intricate interactions between rice and endophytes. Some studies have revealed the saline-alkali resistance mechanism in rice [47,48], but few studies have focused on the mechanism of Na_2_CO_3_ treatment resistance in rice under endophyte infection. We believe that it is valuable to analyze and study the transcriptome and metabolome of endophyte-infected rice leaves under Na_2_CO_3_ treatment. In this experiment, DEGs are involved in starch and sucrose metabolic pathways, and alanine, aspartate, and glutamate metabolic pathways have rarely been reported in previous studies. We believe that these DEGs and DAMs will help us better study and understand the molecular mechanisms underlying the reactions of endophyte-infected rice seedlings to Na_2_CO_3_ treatment.

## 4. Materials and Methods

### 4.1. Endophyte Culture and Rice Treatment

Endophyte EF0801 is a fungus provided by the Microbiology Laboratory, College of Life Sciences, Shenyang Normal University (Shenyang, China). Endophyte EF0801 was isolated and screened from *Suaeda salsa* leaves growing on Panjin’s Red Beach, which can improve plant saline-alkali tolerance and was identified to be 99% similar to *Sordariomycetes* sp. [28]. Endophyte EF0801 was transferred to 125 mL of potato dextrose agar (PDA) medium and placed on the shaker at 26 °C and 120 rpm for 9 days [49]. The culture can be used to treat rice seedlings after successful cultivation.

The experimental material was Liaoxing No. 1 rice, a major cultivar in Liaoning Province, provided by the Rice Crops Research Institute of Shenyang Agricultural University (Shenyang, China). First, healthy seeds were chosen and soaked in 1% sodium hypochlorite for 0.5 h, then washed with purified water. The 100 seeds were cultivated in a beaker containing 750 mL of Hoagland nutrient solution and incubated in a light incubator (28 °C for 16 h day/26 °C for 8 h night, light intensity 10,000 Lux, and relative humidity 80%). According to our previous research [16,18], 10 mM Na_2_CO_3_ concentration was selected to treat rice seedlings in this study. Rice seedlings were cultured for five days and then divided into two groups. Na_2_CO_3_ stressed group (N): cultured in Hoagland solution containing 10 mM Na_2_CO_3_; Na_2_CO_3_ stressed and endophyte EF0801-infected group (EN): cultured in Hoagland solution containing 5% fermentation broth and 10 mM Na_2_CO_3_. Based on the methodology of Liu and Chen [50], it was determined that more than 90% of the rice seedlings were endophyte-infected. On day 7 after the stress treatment, rice seedlings were sampled for further experiments.

### 4.2. Determination of Growth Parameters

Three replicates were established for both the N and EN groups, with 10 seedlings randomly selected from each replicate to measure plant height and root length.

### 4.3. Determination of Sucrose Metabolism-Related Enzyme Activities

SS and SPS activities were determined using the method of Zhang et al. [51]. The leaf sample was homogenized in HEPES-NaOH buffer (50 mM, pH 7.5) and then centrifuged at 14,340× *g* for 10 min at 4 °C to obtain the enzyme extract. The mixture contained supernatant, HEPES-NaOH buffer (50 mM; pH 7.5), MgCl_2_ (50 mM), Fructose (10 mM), and UDPG (10 mM), which was incubated at 37 °C for 30 min. The reaction was stopped by adding 30% NaOH. The activity of SS was determined spectrophotometrically at 480 nm. SPS activity was determined in the same way as SS, except that fructose 6-phosphate was used instead of fructose in the mixture. The activity of SPS was determined spectrophotometrically at 480 nm.

α-Glucosidase (α-GC), β-Glucosidase (β-GC), and Hexokinase (HXK) activities were determined by using α-Glucosidase, β-Glucosidase, and Hexokinase kits (Suzhou Grace Biotechnology Co., Ltd. Suzhou, China)

### 4.4. Metabolome Analysis

Rice leaves of 30 mg were transferred to a 2 mL Eppendorf (EP) tube and extracted with 0.9 mL of extraction solution (V_Methanol_:V_H2O_ = 3:1). In addition, 0.02 mL of ribitol was added as an internal quantitative standard. The sample was ground in a grinder at 50 Hz for 4 min, and then sonicated in an ice water bath for 10 min, and this process was repeated 3 times. Then, the sample was centrifuged at 14,340× *g* for 15 min at 4 °C. Next, 200 μL of the supernatant was transferred into a 1.5 mL EP tube, 40 μL of methoxyamine hydrochloride was added, and the sample was dried under vacuum at 80 °C for 2 h. After that, 60 μL of BSTFA reagent (1% TMCS, *v*/*v*) was added to the sample aliquots and incubated for 1.5 h at 70 °C. All samples were analyzed using an Agilent 7890 GC-MS (Santa Clara, CA, USA). The mass spectrometry data were acquired in full-scan mode with the *m*/*z* range of 50–500 at a rate of 20 spectra per second after a solvent delay of 6.27 min.

The data were processed using ChromaTOF software (V 4.3x, LECO, St. Joseph, MI, USA) and subsequently analyzed for metabolite data using SMICA 14.1 software, including principal component analysis (PCA), partial least squares discriminant analysis (PLS-DA), and orthogonal partial least squares discriminant analysis (OPLS-DA). DAMs were screened for Log_2_FC ≥ 1, *p* < 0.05 and VIP > 1. DAMs were projected to the MetaboAnalyst 6.0 websites (https://www.metaboanalyst.ca/, accessed on 18 March 2024) and KEGG (https://www.kegg.jp/, accessed on 10 March 2024) database to identify the metabolic pathways involved.

### 4.5. Transcriptome Analysis

Total RNA was extracted from frozen rice leaves of the N and EN groups using the RNA Simple Kit (TIANGEN Bio Beijing Co., Ltd. Beijing, China). The purity, concentration, RNA integrity, and quality inspection were examined using a Qubit RNA IQ and Qubit Flex fluorometer (Thermo Fisher Scientific Inc., Waltham, MA, USA). A cDNA library was obtained by PCR enrichment, and the 6 gene expression libraries were named N1, N2, N3, EN1, EN2, and EN3. Finally, sequencing was performed with the Illumina HiSeq platform (Biomarker Technologies Co. Ltd, Beijing, China).

Mapping high-quality clean reads to the *Japonica* rice genome database (https://rapdb.dna.affrc.go.jp/, accessed on 13 January 2024) was conducted using HISAT2 (version 2.0.4). String Tie (FPKM = [total exon fragments/mapped reads (millions) × exon length (kb)]) was used to calculate the mRNA expression level. DESeq2 (version 1.6.3) was used for differential expression analysis, and genes with a false discovery rate (FDR) < 0.05 and |Log_2_FC| ≥ 0.585 were considered DEGs. GO enrichment and KEGG enrichment analyses of DEGs were performed using the GOseq R software package (version 1.34.1), KOBAS website, and KEGG database. Statistical tests for GO and KEGG analyses were performed using Fisher’s method, and *p* ≤ 0.05 was considered a significant change.

### 4.6. Quantitative Real-Time PCR Analysis

Expression levels of endophyte-infected and control rice leaves under Na_2_CO_3_ treatment were examined using qRT-PCR to verify the accuracy of the transcriptome data and the indices of sucrose metabolism-related enzyme activities. Specific primers (Tables S5 and S6) were designed through the Primer3plus website (https://www.primer3plus.com/, accessed on 25 April 2024) (Tables S1 and S2). Total RNA was synthesized into complementary DNA (cDNA) using a PrimeScript RT kit (Takara Bio Inc., Shiga, Japan) containing gDNA Eraser. The reaction mixture contained 4 μL of 5× PrimeScript Buffer, 1 μL of PrimeScript RT Enzyme Mix I, and 1 μL of gDNA Eraser. Reaction conditions are as follows: 37 °C for 15 min and 85 °C for 5 s to inactivate the reverse transcriptase. The qRT-PCR run was conducted on the LightCycler96 PCR system (Roche Co., Ltd., Basel, Switzerland), using SYBR Green I as the fluorescent dye. Each 20 μL reaction mixture contained 10 μL of 2× SYBR Green I Master Mix, 2 μL of diluted cDNA, 0.8 μL of PCR forward primer (10 μM), 0.8 μL of PCR reverse primer, and 6.4 μL nuclease-free water. The qRT-PCR cycling conditions were as follows: initial denaturation at 95 °C for 10 min, 40 cycles at 95 °C for 15 s each, and 60 °C for 1 min. The specificity of the amplification was confirmed by melting curve analysis. In addition, 18s rRNA was quantified as an internal control, and 2^−ΔΔCT^ was used to calculate the relative expression of genes.

### 4.7. Data Analysis

Significance was analyzed using a two-tailed Student’s *t*-test. Statistical analysis was performed using SPSS 29.0, and *p* < 0.05 was considered significant. Results were expressed as mean ± standard deviation (SD).

## Figures and Tables

**Table 1:** Changes in metabolite contents of endophyte-infected versus control rice seedlings under Na_2_CO_3_ stress.

Metabolite	Name	N	EN	FC
Glycolysis and	l-Malic acid	123.43 ± 8.37	216.20 ± 15.91	0.81
TCA cycles	Glucose 6-phosphate	0.49 ± 0.13	0.76 ± 0.10	6.52
	Fructose 6-phosphate	1.81 ± 0.43	2.49 ± 0.21	0.46
	Cis-Aconitic acid	0.15 ± 0.03	0.21 ± 0.03	0.43
	Alpha-Ketoglutarate	25.09 ± 5.94	40.39 ± 11.26	0.68
	Citric acid	729.92 ± 66.67	627.08 ± 59.73	−0.21
Amino acid	l-Asparagine	3.18 ± 0.74	0.00 ± 0.00	−14.8
	l-Glutamate	38.99 ± 6.88	11.07 ± 3.21	−1.82
	l-Aspartic acid	4.43 ± 0.89	3.36 ± 1.96	−0.83
	5-Aminopentanoic acid	0.16 ± 0.03	0.71 ± 0.23	1.27
	Pyroglutamic acid	445.79 ± 43.75	281.20 ± 22.41	−0.66
	l-Threonine	95.25 ± 14.58	49.21 ± 11.62	−0.95
	l-Phenylalanine	3.47 ± 0.62	1.73 ± 0.45	−1.01
	3-Aminoisobutanoic acid	0.42 ± 0.02	0.33 ± 0.04	−0.36
	l-Isoleucine	7.95 ± 1.35	5.72 ± 1.32	−0.47
	l-Lysine	2.20 ± 0.26	4.69 ± 2.39	1.09
Sugars and polyols	d-Glucuronic acid	0.23 ± 0.03	0.31 ± 0.07	8.62
	Sucrose-6-phosphate	0.00 ± 0.00	0.09 ± 0.06	6.52
	Threonic acid	1.21 ± 0.22	2.15 ± 0.23	0.83
	Allose	0.48 ± 0.03	0.23 ± 0.08	−1.03
	d-Mannose	146.01 ± 25.61	187.22 ± 43.21	0.34
	d-Fructose	242.96 ± 42.73	357.43 ± 57.83	0.53
	6-Phosphogluconic acid	0.91 ± 0.03	0.78 ± 0.07	−0.22
	2-Deoxygalactopyranose	7.60 ± 2.92	2.12 ± 1.84	−1.84
	d-Threitol	0.10 ± 0.04	0.17 ± 0.03	0.76
	Cellobiose	0.90 ± 0.28	0.54 ± 0.11	−0.74
	d-Xylose	0.09 ± 0.01	0.11 ± 0.02	0.34
	Isomaltose	0.21 ± 0.12	0.66 ± 0.17	1.66
Organic acid	4-Hydroxycinnamic acid	0.10 ± 0.02	0.29 ± 0.09	1.58
	Oxalic acid	368.55 ± 54.49	456.90 ± 13.54	0.31
	Quinic acid	2.62 ± 0.38	3.02 ± 0.30	0.2
	Gentisic acid	0.24 ± 0.02	0.47 ± 0.06	0.98
	Salicylic acid	5.98 ± 0.43	7.11 ± 0.58	0.3
	Protocatechuic acid	0.15 ± 0.03	0.04 ± 0.06	−0.66
	Ribonolactone	00.14 ± 0.03	0.08 ± 0.00	−0.74
	Glutaric acid	0.09 ± 0.03	0.13 ± 0.03	0.72
	4-Aminobutyric acid	95.74 ± 27.83	69.45 ± 23.55	−0.46

Note: Relative concentration and standard deviation increased by 100 times. FC: fold change. The fold changes of metabolites were represented as log_2_(EN/N).

## Data Availability

Data will be made available on request.

## Supplementary Materials

The following supporting information can be downloaded at: https://www.mdpi.com/article/10.3390/plants14101524/s1, Table S1: Mapping of RNA-Seq data of rice seedlings to the reference genome. Table S2: KEGG enrichment analysis of DEGs in endophyte-infected versus control rice seedlings under Na_2_CO_3_ stress. Table S3: Loading of metabolites to the first principal component (PC1) and the second principal component (PC2). Table S4: KEGG enrichment analysis of endophyte-infected versus control rice seedlings under Na_2_CO_3_ stress. Table S5: Primers used for the qRT-PCR. Table S6: Primers for qRT-PCR of sucrose metabolism-related enzymes.
